# Minimizing ischemic and/or rewarming injury during preservation of porcine kidney grafts

**DOI:** 10.1111/eci.70152

**Published:** 2026-01-23

**Authors:** Charlotte von Horn, Laura Malkus, Thomas Minor

**Affiliations:** ^1^ Surgical Research Department University Clinic of Surgery Essen Germany

## 1 INTRODUCTION

Reperfusion injury remains one of the major problems associated with the preservation and transport of kidney grafts in the setting of renal transplantation.^1^

The pressing shortage of donor grafts has further urged the additional use of organs donated after cardiac arrest of the donor in order to better comply with the number of patients waiting for a new kidney. However, organs that suffered from warm ischemia during retrieval are often conflicted with a reduced functional reserve and may eventually be affected by an even higher risk of graft malfunction after transplantation.^1^

As a consequence, modern preservation techniques have come into practice like continuous hypothermic machine perfusion (HMP) of the endangered kidney graft, that successfully mitigated ischemic preservation injury during transport from donor to recipient. In many countries, renal grafts from extended criteria donors (ECD), including grafts donated after cardiac death (DCD), are now routinely transported by HMP.^2^ However, another issue, besides ischemic preservation injury, that notably affects graft integrity after transplantation is represented by the temperature paradox encountered during reperfusion. Although prerequisite for restitution of physiological homeostasis, the rise in temperature upon reperfusion may result in cellular alterations and mitochondrial dysfunction, if effectuated at exaggerated velocity.^3^

This rewarming injury, in turn, can be largely mitigated by controlling the rewarming process of the tissue by means of oxygenated machine perfusion with an adapted rise of the perfusate temperature.^4^, ^5^

In the present study, the respective contribution of both mechanisms, that is, preservation injury or rewarming injury, in renal reperfusion injury should be investigated.

Therefore, HMP as a tool to reduce ischemic preservation injury and controlled oxygenated rewarming (COR) as a means to mitigate rewarming injury should be compared in an isolated renal perfusion model with special focus on putative additive aspects of HMP and COR.

## 2 MATERIALS AND METHODS

**FIGURE 1:**
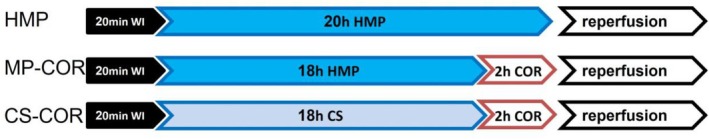
Schematic representation of experimental groups: Hypothermic machine perfusion (HMP), Hypothermic machine perfusion followed by controlled oxygenated rewarming (MP COR), and static cold storage followed by COR (CS‐COR). For details see text.

Porcine kidneys were explanted 20 min after cardiac arrest of the donor and randomized to one of the following protocols (cf. Figure 1).

In the first group, grafts were subjected to oxygenated hypothermic machine perfusion (HMP) at 4°C with University of Wisconsin machine perfusion solution for 20 h at a mean arterial pressure of 30 mmHg.

Other grafts were subjected to 16 h of HMP and 2 h of controlled oxygenated rewarming (MP‐COR) according to a previously established protocol.^6^ To this purpose, grafts were transferred to another perfusion device and perfused with a mixture of 0.5 L Steen solution (XVIVO Perfusion, Göteborg, Sweden) and 0.5 L Ringer's solution to which were added 10 mL sodium bicarbonate 8.4%, 3, 7 mL calcium gluconate 10%, 0.5 g ampicilline and 4 mg of dexamethasone. The temperature of the perfusate was slowly elevated during ongoing perfusion from 10°C to 17°, 30° and 35°C after 30, 60, 75, and 90 min, respectively. Perfusion pressure was adapted in parallel from 30 mmHg up to 75 mmHg during the final steady state period at 35°C solution.

A third group was subjected to 16 h of cold storage prior to COR (CS‐COR). Viability of all grafts was assessed thereafter by warm reperfusion in vitro.

Details on technical methods and statistical analyses are provided as a supplement in the Supporting Information.

## 3 RESULTS

There were no differences regarding individual kidney weight between the groups (HMP: 73.8 ± 6.9 g; MP‐COR: 71.4 ± 4.4 g; CS‐COR: 72.9 ± 7.8 g).

Mean urine production upon reperfusion did not show significant differences between the three groups, although slightly higher values were disclosed in the two groups subjected to COR prior to reperfusion (HMP: 310 ± 142 mL/90 min; MP‐COR: 440 ± 84 mL/90 min; CS‐COR: 454 ± 127 mL/90 min).

Renal perfusate flow upon reperfusion remained rather constant during the reperfusion period in all groups. However, absolute values differed according to whether controlled rewarming was applied prior to reperfusion or not: Mean values were 138 ± 34 mL/min after HMP, 239 ± 47* mL/min after MP‐COR, and 288 ± 89* mL/min after CS‐COR (*: *p* < 0.05 vs. HMP).

No relevant difference could be seen between MP‐COR or CS‐COR.

**FIGURE 2:**
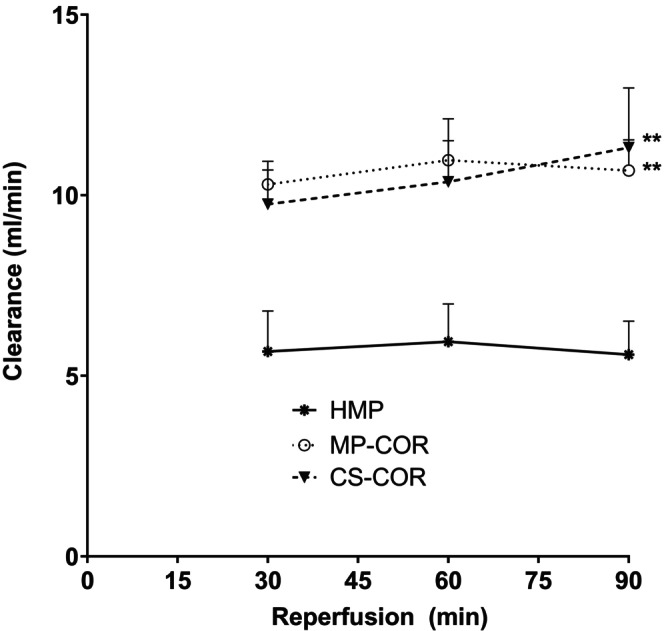
Renal clearance of creatinine upon reperfusion after preservation by hypothermic machine perfusion (HMP), HMP followed by controlled oxygenated rewarming (MP‐COR) or static cold storage followed by COR (CS‐COR). (*: *p*<0.01 vs. HMP; comparison of area under the curve by ANOVA and Tukey‐test)

Glomerular filtration was similarly affected by the preceding preservation protocol (cf Figure 2). HMP resulted in stable clearance rates over the entire observation period of around 5 mL/min. By contrast, renal clearance of creatinine was seen significantly increased to nearly twice the value if COR was applied prior to reperfusion. Most interestingly, this effect of COR was similar, even when only static cold storage was used during preceding preservation.

**FIGURE 3:**
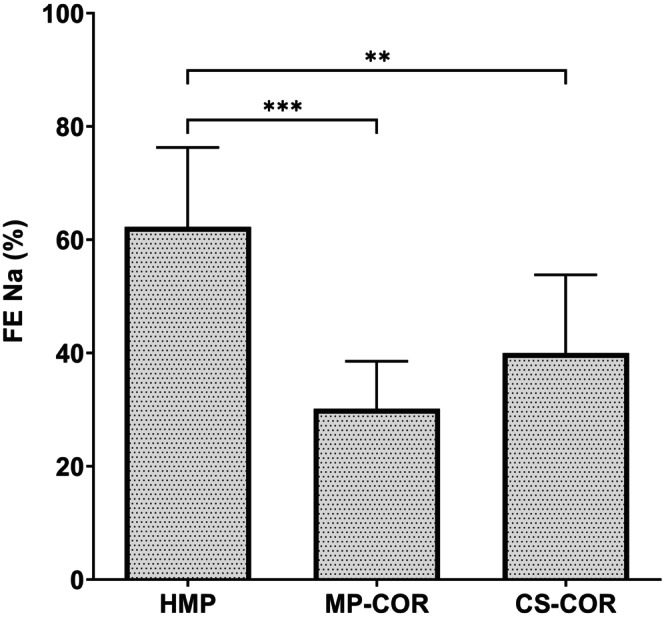
Fractional excretion of sodium (FENa) upon reperfusion after preservation by hypothermic machine perfusion (HMP), HMP followed by controlled oxygenated rewarming (MP‐COR), or static cold storage followed by COR (CS‐COR). (***: *p*<0.001; ***: *p*<0.01 vs. HMP by ANOVA and Tukey‐test)

Tubular cell function was evaluated by the fractional excretion of sodium during reperfusion (FENa) (cf. Figure 3).

It was seen that FENa turned out to be significantly reduced, if COR was added to the HMP protocol, indicating that tubular reabsorption of sodium was positively affected by the controlled rewarming at the end of hypothermic perfusion.

Moreover, static preservation and COR (CS‐COR) also resulted in significantly better tubular cell function than preservation by HMP without COR.

The ratio of total tubular reabsorption of sodium (as the major source of renal energy consumption) and the corresponding oxygen consumption rate (VO_2_) pretty well reflects the efficiency of renal mitochondrial oxygen utilization,^7^ which has been considered a key target of rewarming injury.^8^, ^9^

**FIGURE 4:**
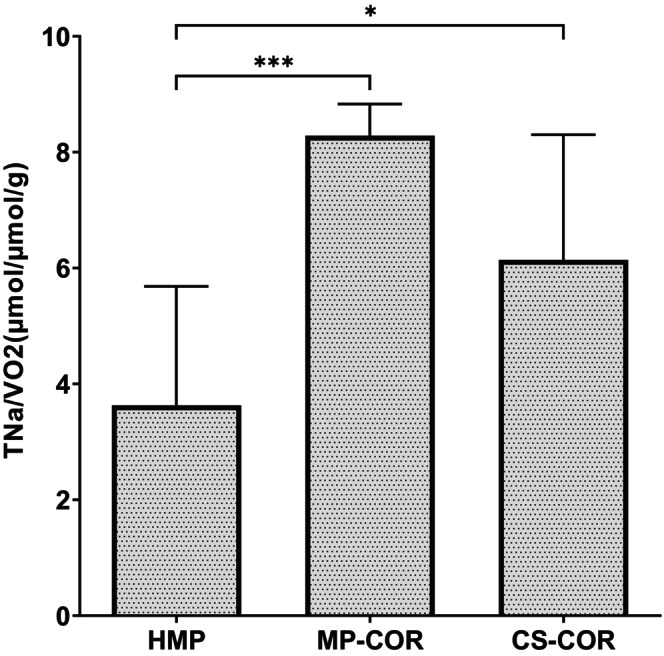
Efficiency of oxygen utilization (ratio of total sodium absorption and oxygen consumption‐TNa/VO2) upon reperfusion after preservation by hypothermic machine perfusion (HMP), HMP followed by controlled oxygenated rewarming (MP‐COR), or static cold storage followed by COR (CS‐COR). (*: *p*<0.05, ***: 0.001 vs. HMP by ANOVA and Tukey‐test).

At the conclusion of the experiment, the ratio of TNa/VO_2_ was calculated and disclosed a significant improvement in renal efficiency of oxygen utilization if HMP was complemented by a controlled oxygenated rewarming prior to reperfusion (cf. Figure 4).

**FIGURE 5:**
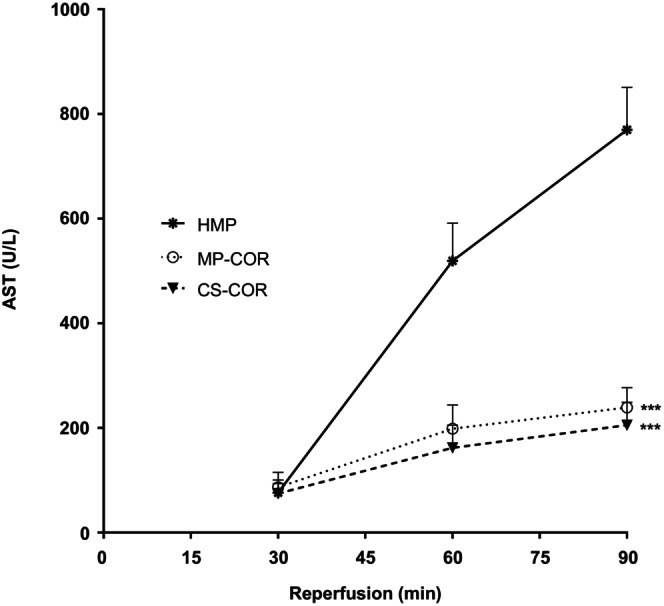
Perfusate activities of aspartate aminotransferase (AST) upon reperfusion after preservation by hypothermic machine perfusion (HMP), HMP followed by controlled oxygenated rewarming (MP‐COR) or static cold storage followed by COR (CS‐COR). (***: *p*<0.001 vs. HMP, comparison of area under the curve by ANOVA and Tukey test)

Structural tubular cell injury was also affected by the preservation protocol. Renal release of AST into the perfusate upon reperfusion is depicted in Figure 5. Although initial perfusate activities of AST were quite similar in all groups, a steep rise was noted during ongoing reperfusion in the HMP group, which was significantly attenuated by COR, independent of whether preceding hypothermic preservation was effectuated by HMP or CS.

Total release of HMGB1, as a danger‐associated pattern pertinent to the innate immune signalling, was also influenced by the preservation protocol.

While COR after HMP was effective in significantly reducing perfusate concentrations of HMGB1 in comparison to HMP alone, COR after cold storage did not result in a comparable reduction of the danger‐associated pattern (77.5 ± 11.1 vs. 33.24 ± 9.9 vs. 56.5 ± 35.0 pg/mL; HMP vs. MP COR vs. CS‐COR, resp.; ** *p* < 0.01 vs. HMP).

## 4 DISCUSSION

Most repercussions of ischemic tissue alterations do not manifest during preservation but only during subsequent reperfusion.^8^ The unhealthy dys‐homeostasis incurred during organ preservation is often reversible but prone to trigger reperfusion injury during restoration of physiological metabolic conditions.

Thus, changing the modalities of reperfusion offers a therapeutic means to reverse unhealthy priming and mitigate reperfusion injury.^3^

Controlled elevation of tissue temperature by thermally graduated machine perfusion prior to normothermic reperfusion, for instance, has proven an effective tool to reduce ‘rewarming injury’ and to enhance ulterior renal function in experimental models.^5^, ^9^ Clinical experience with this technique known as controlled oxygenated rewarming (COR) is more scarce, but existing data are promising.^10^, ^11^

The results of the present study suggest that COR provides a significant added benefit to renal grafts preserved by oxygenated HMP. Besides an improvement in glomerular filtration rate, COR also positively affected tubular reabsorption of sodium. Elevated fractional excretion of sodium is known to be indicative of tubular damage, a frequent repercussion of preservation/reperfusion injury.^12^ Energy‐dependent transport of electrolytes, especially sodium, across the renal tubular cells accounts for the vast majority of renal energy expenditure and can be taken as an indicator of the efficiency of mitochondrial oxygen utilization when set into rapport with overall oxygen consumption of the isolated perfused kidney.

Impairment of the respiratory chain at the mitochondrial level has already been shown to be triggered by cold preservation of the kidney^13^ and to be severely aggravated by abrupt rewarming upon reperfusion^13^ even after preceding oxygenated perfusion at lower temperatures.^14^ The aggravation of mitochondrial uncoupling, incurred during abrupt warming up of hypothermic kidneys, can, however, be counteracted by COR, thereby also significantly enhancing spare respiratory capacity.^3^, ^9^

Accordingly, Ogurlu and co‐workers^15^ have disclosed a significantly enhanced restoration of tissue levels of ATP if controlled rewarming was applied after cold preservation of porcine kidneys by HMP. However, unlike our results, the protective effects of COR on other parameters, like FENa or glomerular filtration rate were less consistent in their study.

In contrast to us, they apparently used a rather steep rise of the temperature already during the transition from 10°C to 20°C, while our protocol follows a sigmoidal curve that is particularly flattened at the beginning of the procedure.^6^

As rewarming injury is thought to take place primarily during transit between 10°C and 20°C,^16^ but is hardly seen at temperatures above 16°C,^17^ the possibly better adapted temperature curve might have accounted for the more consistent results of the present investigation.

Besides the clear observation that controlled rewarming prior to reperfusion provides a significant additive benefit to renal preservation by HMP, our data also indicate that.

COR without HMP turns out to yield better functional results than HMP without COR.

Thus, the contribution of hypothermic preservation to the tissue damage seems outweighed by the cellular injury triggered during the abrupt rewarming upon reperfusion.

From here, it seems tempting to expand the focus of therapeutic measures to cope with graft injury during transplantation towards the critical period of initial reperfusion and rewarming.

Conclusions derived from the present study are to be drawn under the restriction that any experimental model is prone to certain limitations. The isolated porcine kidney had been chosen as a model based on the anatomical and physiological similarities between human and porcine organs. The size of a large animal organ corresponds satisfactorily to the conditions encountered in a clinical setting, and the technical equipment is the same for both. Unlike an in vivo model including actual transplantation of the graft, the in vitro approach recommends itself as a screening model for first‐line physiological research and corresponds very well to the three ‘r’ regulations of most directives on animal research.

It allows for a technically easy and reproducible measurement of initial functional recovery of the kidney. Besides short‐term follow‐up of glomerular filtration rate, central parameters of our research goal, like the analysis of total sodium absorption rate and oxygen utilization efficiency, are accessible.

The limits inherent to this study design should not be neglected. Thus, data on long‐term renal function cannot be provided by in vitro approaches, and the relatively small number of replicates in each group cannot compare to clinical research trials. On the other hand, the controlled circumstances of the in vitro protocol as well as the standardized conditions of the individual kidneys are aspects in favor of a valid conclusion.

Taken together, our data point out that optimization of hypothermic kidney preservation, for example, by oxygenated machine perfusion, does immunize against rewarming injury upon reperfusion.

By contrast, pro‐active mitigation of rewarming injury by controlled oxygenated rewarming prior to transplantation seemingly provides better protection of immediate renal function, even if performed after conventional organ preservation by static storage.

## AUTHOR CONTRIBUTIONS

**Charlotte von Horn** and **Thomas Minor**: Research design. **Charlotte von Horn**: Data analysis. **Laura Malkus**: Performance of research. **Charlotte von Horn** and **Thomas Minor**: Writing—original draft preparation. **Laura Malkus**: Writing—review and editing:

## FUNDING INFORMATION

This research was supported by institutional funds.

## CONFLICT OF INTEREST STATEMENT

The authors declare no conflicts of interest.

## Supporting information


Data S1.
