# Supplementary material for: Minimizing ischemic and/or rewarming injury during preservation of porcine kidney grafts
Source: Eur J Clin Invest. 2026 Jan 23;56(1):e70152. doi: 10.1111/eci.70152 (PMC12829302; doi:10.1111/eci.70152)
Supplement: Supplementary file 1 — Data S1. [file ECI-56-e70152-s001.docx]

**Materials and Methods**

*Organ retrieval and study design*

Animals were treated according to the rules and after approval of the local authorities. The federal law regulating the protection of animals and the principles of laboratory animal care (NIH publication vol 25, No 28, revised 1996) were followed.

All experiments were carried out only on isolated organs and in accordance with the EU Directive 2010/63/EU for animal experiments.

Kidneys were procured after cardiac arrest from healthy German Landrace pigs that died during retrieval of the liver for another scientific project, approved by the responsible authorities (Landesamt für Natur, Umwelt und Klima -LANUK), NRW, Germany (AZ 81-02.04.2023.A285).

Twenty-one kidneys from female German Landrace/Pietrain crossbred pig, aged between 12 and 14 weeks, were used for this project.

The donor animals were allowed to acclimatize to their surrounding for a minimum of 10 days prior to surgery and had free access to tap water and standard pellet food. They were kept in pairs on a floor with a non-slip epoxy resin coating littered with straw. As enrichment, the animals also receive a hay basket, a permanently mounted wall brush and balls or other chewing equipment in regular rotation per box. The animals were kept at a room temperature of 22°C. Solid food was withdrawn 20 hours before surgery.

After intramuscular pre-medication with tiletamin / zolazepam (4 mg/kg) an ear vein was cannulated and general anesthesia induced by intravenous injection of propofol (3 mg/kg) followed by intubation and mechanical ventilation with isoflurane (1.5-2%) in air/oxygen.

Under general anesthesia and continuous infusion of fentanyl (0.015 mg/kg/h), the liver was dissected free and the animals were euthanized by intra-cardiac injection of KCl. The kidney was removed 20 min after induction of cardiac arrest. No heparin was given prior to organ retrieval. Following isolation of the graft, the kidney was flushed on the back-table with HTK solution from a height 100 cm till the effluent was clear. All kidneys were randomly assigned to one of the following groups (cf. figure 1):

1. Hypothermic machine perfusion (HMP): Grafts were subjected to an oxygenated hypothermic machine perfusion at 4°C with University of Wisconsin Machine perfusion solution (Bridge to life Ltd, Columbia, SC) for 20h at a mean arterial pressure of 30mmHg.
2. Machine perfusion+controlled oxygenated rewarming (MP-COR): Grafts were put on oxygenated hypothermic machine perfusion at 4°C like in group HMP for 18h but then subjected to 2h of controlled oxygenated rewarming according to a previously established protocol ^1,2^. To this purpose, grafts were transferred to another perfusion device and perfused with a mixture of 0.5 l SteenTM solution (XVIVO Perfusion, Göteborg, Sweden) and 0.5 l Ringer’s solution to which were added 10 ml sodium bicarbonate 8.4%, 3,7 ml calcium gluconate 10%, 0.5 g ampicilline, and 4 mg of dexamethasone. The temperature of the perfusate was slowly elevated during ongoing perfusion from 10°C to, 17°, 30° and 35°C after 30, 60, 75 und 90 min, respectively ^3^. Perfusion pressure was adapted in parallel from 30mmHg up to 75mmHg during the final steady state period at 35°C solution.
3. Cold storage+controlled oxygenated machine perfsuion (CS-COR): Grafts were statically preserved overnight in HTK solution for 18 h at a temperature of 4°C regulated by a cryo-thermostat. Thereafter the kidneys were subjected to 2h of controlled oxygenated rewarming as described above for group MP-COR.

*Reperfusion model*

Graft integrity was tested by isolated reperfusion in vitro using an established model as described earlier ^4,5^.

In brief: 1000 ml freshly prepared Krebs-Henseleit buffer containing 2.2% bovine serum albumin, 50mg creatinine and 20 ml of concentrated amino acid solution (RPMI 1640-50x) was recirculated at 37°C through the graft with a perfusion pressure of approx. 90mmHg maintained by a servo-controlled roller pump.

The perfusate was oxygenated by a hollow fibre oxygenator and the kidney placed in a temperature regulated moist chamber. Urine was collected via PE-tubing inserted into the ureter and re-infused to the reservoir every 30 min ^6^ ^7^.

Before reperfusion, all organs were rinsed with 100 ml of cold saline solution and exposed to no flow conditions at room temperature for 20 min in order to imitate warm ischemia time during surgical implantation in the clinical setting ^8^.

*Measurements*

Concentrations of creatinine were determined in perfusate and corresponding urine samples by reflectance photometry on an Element RC3X point of care unit (scil animal care company, Viernheim, Germany). Clearances were calculated for the respective intervals as urinary creatinine x urine flow (ml/min) / perfusate creatinine.

Oxygen partial pressure in arterial and venous perfusate samples as well as sodium levels in perfusate and urine were measured using an acid base laboratory (ABL800 flex, Radiometer, Copenhagen, Denmark).

# Fractional excretion of sodium (FE Na) has been calculated according to:

# FE Na= Na_(urine)_ x Creatinine_(perfusate)_ / Na_(perfusate)_ x Creatinine_(urine)_ x 100.

# The efficiency of renal O_2_ utilization was approximated by the ratio of total kidney transport of Na (TNa), - accounting for the vast majority of energy consuming processes in the kidney ^9^, and VO_2_, with TNa being equal to filtered Na minus excreted Na: TNa = (GFR x Perfusate Na) – (urinary Na x urine flow)

Renal release of aspartate aminotransferase (AST) was taken as a global indicator of cellular damage upon reperfusion and quantified in the circulating perfusate by reflectance photometry.

High mobility group box-1 (HMGB1) released into the perfusate was quantified using porcine ELISA tests from Cloud-Clone Corp., Wuhan, China according to the manufacturer’s instruction.

*Statistics*

All values are expressed as means±SD of n=7 experiments per group.

After proving the assumption of normality, differences between the groups were tested by one way ANOVA and post hoc testing with the Tukey Kramer test, or nonparametric comparison using Kruskal-Wallis Statistic, when necessary (Instat 3.01;Graph Pad software Inc, San Diego, CA), unless otherwise indicated. Statistical significance was set at P<0.05.

1. Minor T, von Horn C, Gallinat A, et al. First-in-man controlled rewarming and normothermic perfusion with cell-free solution of a kidney prior to transplantation. *Am J Transplant.* 2020;20(4):1192-1195.

2. von Horn C, Zlatev H, Kaths M, Paul A, Minor T. Controlled Oxygenated Rewarming Compensates for Cold Storage-induced Dysfunction in Kidney Grafts. *Transplantation.* 2022;106(5):973-978.

3. Zlatev H, von Horn C, Kaths M, Paul A, Minor T. Clinical use of controlled oxygenated rewarming of kidney grafts prior to transplantation by ex vivo machine perfusion. A pilot study. *Eur J Clin Invest.* 2022;52(2):e13691.

4. Minor T, von Horn C. Reduction of Renal Preservation/Reperfusion Injury by Controlled Hyperthermia During Ex Vivo Machine Perfusion. *Clin Transl Sci.* 2021;14(2):544-549.

5. Zlatev H, von Horn C, Minor T. Preservation of Mitochondrial Coupling and Renal Function by Controlled Oxygenated Rewarming of Porcine Kidney Grafts. *Biomolecules.* 2021;11(12):1880.

6. von Horn C, Wilde B, Rauen U, Paul A, T M. Use of the new preservation solution Custodiol-MP for ex-vivo reconditioning of kidney grafts. *Transplant International.* 2020;33(S2):34-35.

7. Weissenbacher A, Huang H, Surik T, et al. Urine recirculation prolongs normothermic kidney perfusion via more optimal metabolic homeostasis-a proteomics study. *Am J Transplant.* 2021;21(5):1740-1753.

8. Minor T, Yamaguchi T, Isselhard W. Effects of taurine on liver preservation in UW solution with consecutive rewarming in the isolated perfused rat liver. *Transplant Int.* 1995;8:174-179.

9. Pei L, Solis G, Nguyen MT, et al. Paracellular epithelial sodium transport maximizes energy efficiency in the kidney. *J Clin Invest.* 2016;126(7):2509-2518.
